# An optimal glycemic load range is better for reducing obesity and diabetes risk among middle-aged and elderly adults

**DOI:** 10.1186/s12986-020-00504-5

**Published:** 2021-03-22

**Authors:** Fengyi He, Chaogang Chen, Feng Li, Yiqin Qi, Xiuhong Lin, Ping Liang, Meng Ren, Li Yan

**Affiliations:** 1 Department of Clinical Nutrition, Sun Yat-Sen Memorial Hospital of Sun Yat-Sen University, Guangzhou, 510120 China; 2 Department of Endocrinology, Sun Yat-Sen Memorial Hospital of Sun Yat-Sen University, Guangzhou, 510120 China

**Keywords:** Cluster analysis, Glycemic load, Diabetes, Obesity

## Abstract

**Background:**

Due to the lack of evidence, advice pertaining to glycemic load (GL) can be misleading. Does the excessive restriction of GL, mostly through an extreme reduction in carbohydrate intake, result in a relatively high intake of fat and protein and result in overweight and obesity? This study was performed to initially explore the optimal GL range.

**Methods:**

A cross-sectional study involving 2029 participants aged 40 years or older in Guangzhou, China was conducted. Participants were divided into four groups according to cluster analysis. Dietary data were assessed using a previously validated 3-day food record.

**Results:**

Instead of participants with the highest [cluster 1, median (interquartile ranges) GL was 112(107–119)/1000 kcal] and the lowest GL intake [cluster 4, 90(82–96)/1000 kcal], those with moderate GL intakes [clusters 2 and 3, 93(85–102) and 93(85–99)/1000 kcal, respectively] had a lower prevalence of overweight, obesity and diabetes. In addition, clusters 2 and 3 were more consistent with the macronutrient intake reference with adequate micronutrient intake. Therefore, the optimal GL range was determined to be (85–100)/1000 kcal, rather than “lower is better”.

**Conclusions:**

Reducing the GL intake to prevent diabetes deserves more attention in the context of a balanced diet. An appropriate GL may be better than excessive restriction.

## Introduction

The steady increase in the prevalence of diabetes over the past three decades in virtually all regions worldwide has received considerable interest; the number of people with diabetes in 2017 was 425 million, and this figure is predicted to increase to 629 million by 2045 [1]. In general, the prevalence of diabetes in China has sharply increased; the prevalence has been reported to be less than 1% in 1980 [2], 5.5% in 2001 [3], 9.7% in 2008 [4], 11.6% in 2010 [5], and 10.9% in 2013 [6]. Most diabetic patients suffer from type 2 diabetes, which is mostly avoidable. The most important modifiable risk factors for type 2 diabetes are overweight and obesity, an improper diet, a sedentary lifestyle and tobacco smoking [7].

Numerous observational studies and clinical trials have investigated the role of nutrition in the prevention of diabetes. With respect to macronutrients, it has been shown that the quality rather than the quantity of carbohydrates is associated with increased diabetes risk [8]. Compared to the traditional view of carbohydrate restriction alone, the glycemic index (GI) was created as a tool to guide people with diabetes in selecting foods [9]. Subsequently, the glycemic load (GL), which considers the GI and the amount of available carbohydrates eaten [10], was introduced and considered the primary measurement of carbohydrate quantity and quality. Reducing the GL may provide a modest additional benefit [11, 12]. Several prospective observational studies [13, 14] and meta-analyses [15, 16] have shown that the diabetes risk increases with a higher dietary GL. In addition, a low-GL diet may have favorable effects in individuals with prediabetes who constitute a broad group with a high risk of developing diabetes [17].

However, traditional association analyses in the field of nutritional epidemiology typically examine disease in relation to a single GL, regardless of the rationality of the energy ratio of macronutrients and food intakes. Although these analyses have been quite valuable, the results of the association between GL, health and disease may be affected. Whether the unsatisfactory effect of a low-GL diet should be attributed to its innate reasons or irrationality of the energy ratio of macronutrients and food intake is uncertain.

Previous studies were interested in indicating the benefit of reducing carbohydrate intake [18]. However, energy intake is derived from carbohydrates, fat and protein. The following question emerged: Can the excessive restriction of GL, mostly through an extreme reduction in the intake of carbohydrates, result in a relatively high intake of fat and protein and result in overweight and obesity [19]? Indeed, China is facing an emerging obesity epidemic, and the prevalence of overweight and obesity has doubled over the past decade [20]. However, few previous studies examining the relationship between dietary GL and diabetes risk considered physiological endpoints, such as obesity.

In addition, definitive data warranting the establishment of evidence-based dietary GL recommendations are currently lacking. Several studies conducted outside of Asia have defined a low-GL diet as the maintenance of a GL less than 80 per day [21], the consumption of no more than 45 per 1000 kcal [22], or the consumption of no more than one serving of high-GL foods per day [23–25]. Such targets are difficult to achieve based on the Chinese Dietary Guidelines. Grains such as rice and noodles form the base of nearly every meal in the Chinese diet. According to the Chinese dietary guidelines, an adult should intake 250–400 g/day of grains, of which 50–150 g should be whole grains or mixed beans and 50–100 g should be tubers. The GL of the recommended diet would be more than 130 per day or 80 per 1000 kcal. Due to the existence of limited or no supporting evidence, advice pertaining to the GL can be misleading. This study, which involved 2029 middle-aged and elderly Chinese adults, was performed to examine the associations among GL, diabetes and obesity while considering the rationality of nutrient intake; in addition, this study initially aimed to determine the optimal GL range.

## Methods

### Study population

This study is an ongoing multiethnic, epidemiological study investigating lifestyle and the glucose metabolism state in China [26–28]. The data used in this cross-sectional analysis were obtained from a baseline survey conducted between July 2011 and December 2011 and focused on a subsample in Guangzhou, China. The details of the study methodology have been previously published [29]. Briefly, all eligible adults who were (a) aged 40 years or older and (b) lived in Guangzhou for at least 3 years were recruited. The participants were excluded if they (a) had a previous diagnosis of diabetes and/or were using oral diabetes medication or insulin injection and/or (b) had a severe impairment in their cardiac, hepatic or renal function.

### Dietary assessment

During the first face-to-face interview, all participants were trained in the level of detail required to adequately describe the foods and amounts consumed, including the name of the food (brand name, if possible), preparation methods, recipes for food mixtures, and portion sizes. Food models and measuring displays were used to ensure accurate portion sizes. Subsequently, the participants were instructed to record the amount and type of all foods and drinks they consumed during a continuous 3-day period, which ideally included 2 weekdays and 1 weekend day at home, highly suggesting that recording to be done at the time of the eating occasion in order to reliance on memory. The foods eaten daily, the brand name, and the food preparation method were recorded in detail. The amounts consumed may be measured using a scale or household measures (e.g., cups or tablespoons) or estimated using models or pictures. All records were received in real time, clarified entries and probed for forgotten foods by a dietitian prior to collection.

The Food Composition Table of China and international GI tables [30, 31] were used to establish a software model to log, calculate and save the energy, nutrients, GI and GL values of the study subjects. An appropriate GI value was chosen based on the cooking method used (e.g. uncooked, boiled or fried). The mean GI value was calculated when multiple values were available. For foods without a published GI value, the GI value was estimated based on a standardized method [32]. The GL of each food was calculated by multiplying the carbohydrate content in each serving by the GI of that food, and the total GL was calculated as the sum of all GL values of each food consumed over the course of 1 day [32]. In addition, for each participant, the energy and nutrient intake were adjusted by the ideal body weight by converting the total value into the value per 1 kg of ideal body weight [33]. The mean daily indices of dietary intake were calculated.

The nutrient adequacy ratios (NARs) were calculated for 14 micronutrients (vitamin A, thiamine, riboflavin, ascorbic acid, folic acid, calcium, potassium, magnesium, iron, zinc, selenium and manganese) by dividing the participants’ actual intakes of each micronutrient by the recommended nutrient intake (RNI) or adequate intake (AI). An NAR equal to 0 indicates a diet devoid of that micronutrient, whereas an NAR equal to 1 indicates a diet that achieved or exceeded the recommended nutrient intake of that micronutrient. To obtain an overall estimate of nutritional adequacy, a mean micronutrient adequacy ratio (MAR) was calculated based on the 14 NARs. Each NAR was truncated at 1 to avoid the possibility that a micronutrient with a high NAR compensates for a micronutrient with a low NAR. Therefore, the maximum possible MAR value was 1, and the minimum possible MAR value was 0.

Reproducibility and validity tests of the 3-day food record were conducted using the answers obtained from 58 participants [34]. These participants completed the 3-day food record and food frequency questionnaire (FFQ) for the first time. Then, after approximately 2 weeks, they completed a second 3-day food record. The Spearman correlation coefficients of the two food records were 0.62 for the GI and 0.65 for the GL and ranged from 0.41 to 0.69 for the GLs of different food groups (*P* < 0.05 for all). Similarly, the Spearman correlation coefficients of the food records and FFQ were 0.54 for GI and 0.42 for GL and ranged from 0.32 to 0.55 for the GLs of different food groups (*P* < 0.05 for all).

### Nondietary exposure assessment

The data regarding the sociodemographic characteristics and lifestyle information, including physical activity, educational history, smoking and alcohol drinking status were gathered by trained interviewers using a standard questionnaire. In addition, the participants were invited to complete an oral glucose tolerance test (OGTT).

Physical activity was expressed as the number of metabolic equivalent hours per week (MET-h/week) [35]. The MET-h of an activity was calculated by multiplying the time spent performing the activity by the MET value corresponding to that activity. Then, the total MET-h/week of moderate to vigorous activities was calculated by adding the MET-h values of different moderate and vigorous activities in a week. Regular exercise was defined as performing at least 7.5 MET-h of moderate to vigorous activities per week.

The participant’s weight, height and waist circumference were measured with the participants dressed in light clothing without shoes in the fasting state. Their body height and waist circumference were measured to the nearest 0.1 cm, and their weight was measured to the nearest 0.1 kg. High-quality and accurate techniques and mean measurements were used. The body mass index (BMI) was calculated as the weight in kilograms divided by the square of the height in meters. Overweight was defined as a BMI between 24.0 and 27.9 kg/m^2^, and obesity was defined as a BMI greater than or equal to 28.0 kg/m^2^. Central obesity was defined as a waist circumference greater than or equal to 85.0 cm for men and 80.0 cm for women.

The plasma glucose level was measured by a glucose oxidase assay (AU5821; Beckman Coulter, Miami, FL, USA). The intra- and interassay coefficients of variation were 2 and 3%, respectively. Peripheral blood samples were collected in the morning after 8–12 h of fasting. The fasting plasma glucose (FPG) and 2-h plasma glucose (2-hPG) levels were measured at fasting and 2 h after the participants had ingested a standard 75-g glucose solution, respectively. Diabetes was defined as an FPG level greater than or equal to 7.0 mmol/L, a 2-hPG level greater than or equal to 11.1 mmol/L, and/or a self-reported diagnosis of diabetes supported by reliable medical reports.

### Data cleaning

The participants were considered fully eligible if it was verified that complete data were adequately recorded. In addition, to prevent the variables with larger ranges from having a greater contribution than the variables with smaller ranges, z-scores were calculated to standardize the data set before clustering. Univariate and multivariate outliers (> 3 SD) were removed.

### Statistical methods

All statistical tests were performed using PASW SPSS Statistics for Windows, Version 18.0 (IBM SPSS, Armonk, NK, USA), and the significance level was set at *P* < 0.05. The continuous variables are expressed as the mean ± SD and were compared using one-way ANOVA followed by Student–Newman–Keuls (SNK) test for post hoc pairwise comparisons. The categorical variables are expressed as absolute values (relative frequencies) and were compared using the chi-squared test.

A dominant component analysis was performed to identify the underlying dietary patterns. Bartlett’s test of sphericity and the Kaiser–Meyer–Olkin (KMO, > 0.60) measure of sampling adequacy were used to verify the appropriateness of the component analysis. The components were also orthogonally rotated (the varimax option) to enhance the difference between loadings, which allowed for easier interpretability. Components were retained based on the eigenvalues > 1.0. A k-means cluster analysis was used to classify the participants into clearly distinct groups based on the dominant components.

The odds ratio (OR) and 95% confidence interval (CI) of the prevalence of disease according to the clusters were assessed by logistic regression. In the fully adjusted model, advanced age (≥ 65 years or no), sex (male or female), regular exercise (yes or no), current smoking status (yes or no), current drinking status (yes or no), and diabetic family history (yes or no) were adjusted.

## Results

### Participant characteristics

**Fig. 1:**
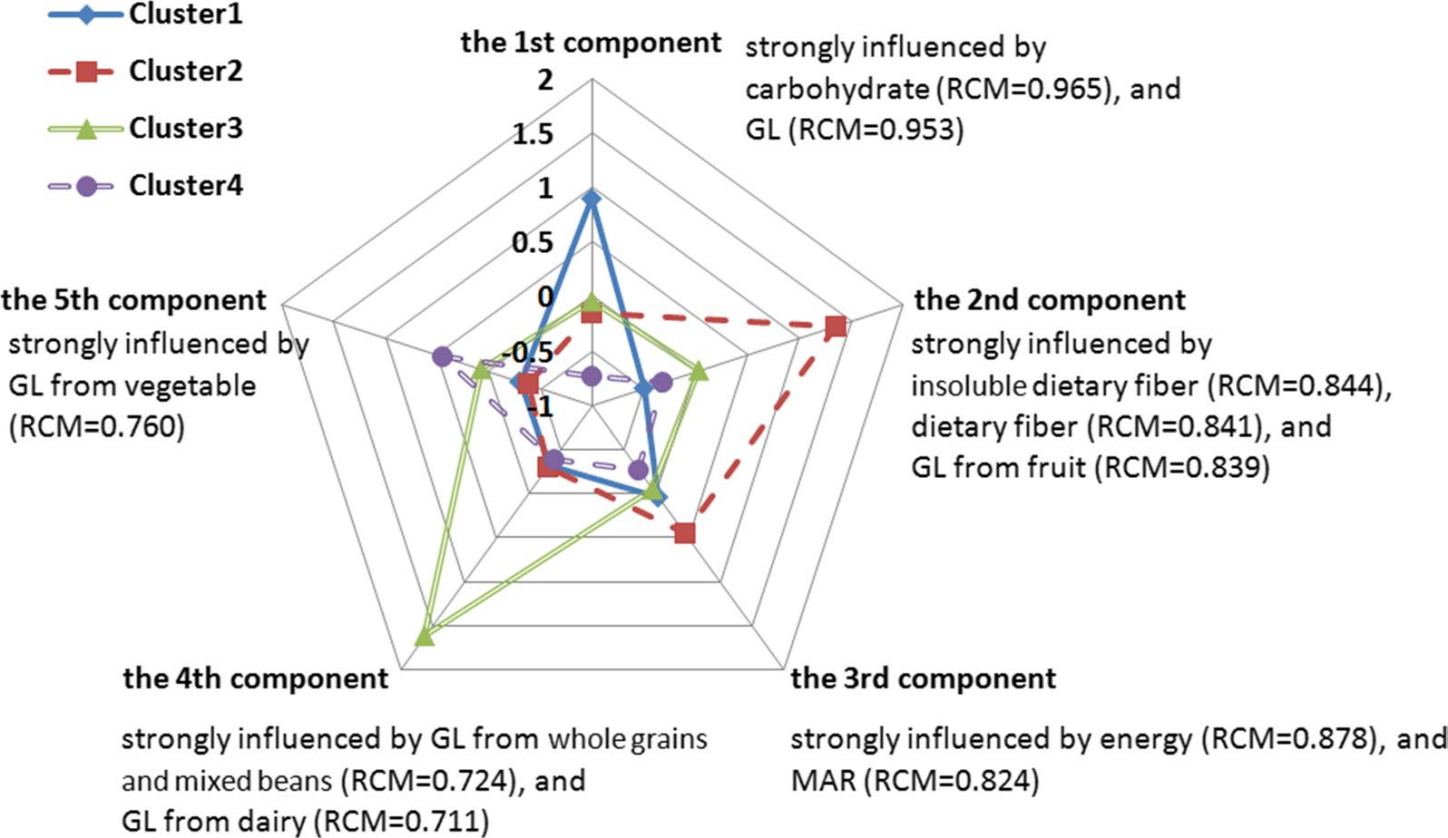
Four identified clusters on dominant component loadings after varimax rotation. *RCM* rotated component matrix, *GL* glycemic load, *MAR* micronutrient adequacy ratio

After excluding outliers and participants with incomplete data, 2029 participants (628 men and 1401 women) with a mean age of 56 years were included in the analysis. Bartlett’s test of sphericity and a KMO of 0.654 supported the appropriateness of the component analysis. Five principal components were extracted through a dominant component analysis of 16 variables, explaining 73.8% of the variance in the model (Fig. 1).

The general, anthropometric, and laboratory characteristics of the participants classified in different clusters are shown in Table 1. Cluster 1 included more male subjects and tended to have an unhealthier lifestyle pattern, such as smoking and less regular exercise. Clusters 2 and 3 included more female subjects and tended to have a healthier lifestyle pattern. Cluster 4 tended to include more younger subjects.

**Table 1:** General, anthropometric, and laboratory characteristics of the study subjects classified in different clusters

	Cluster 1 (n = 627)	Cluster 2 (n = 374)	Cluster 3 (n = 357)	Cluster 4 (n = 671)	$\upchi ^{2} /{\text{F}}$	*P*	$\upvarphi /\upeta$
Advanced age (n (%))	79 (12.6)	40 (10.7)	50 (14.0)	58 (8.6)	8.573	0.036	0.065
Male (n (%))	330 (52.6)	58 (15.5)	62 (17.4)	178 (26.5)	216.606	< 0.001	0.327
Current smoker (n (%))	123 (20.1)	25 (6.9)	17 (4.9)	84 (12.9)	60.632	< 0.001	0.175
Regular alcohol drinkers (n (%))	25 (4.0)	7 (1.9)	9 (2.5)	25 (3.7)	4.426	0.219	0.047
Regular exercise (n (%))	93 (14.8)	88 (23.5)	84 (23.5)	121 (18.0)	17.211	0.001	0.092
Diabetic family history (n (%))	107 (17.1)	59 (15.9)	60 (16.9)	109 (16.4)	0.327	0.955	0.013
BMI (kg/m^2^)	23.3 ± 2.90	23.3 ± 3.0	23.0 ± 3.1	23.5 ± 3.2	1.452	0.226	0.046
Waist circumference (cm)	81.7 ± 8.8^bc^	80.0 ± 8.8^ad^	79.2 ± 8.9^ad^	81.3 ± 9.5^bc^	7.797	< 0.001	0.107
FPG (mmol/L)	5.54 ± 0.96^b^	5.37 ± 0.61^a^	5.49 ± 0.90	5.43 ± 0.86	3.610	0.013	0.073
2-hPG (mmol/L)	7.85 ± 2.77^bc^	7.27 ± 1.89^a^	7.44 ± 2.06^a^	7.60 ± 2.33	5.269	0.001	0.088
AUCG	6.69 ± 1.72^bd^	6.32 ± 1.10^a^	6.47 ± 1.30	6.51 ± 1.44^a^	5.512	0.001	0.090

Categorical variables are expressed as absolute values (relative frequencies) and were compared using the chi-squared test. Continuous variables are expressed as the mean ± SD and were compared using ANOVA and SNK. Advanced age was defined as an age ≥ 65 years; regular exercise was defined as ≥ 7.5 MET-h/w of moderate to vigorous activities

*BMI* body mass index, *FPG* fasting plasma glucose, *2-hPG* 2-hour plasma glucose, *AUCG* area under the curve of glucose

^a^Compared to Cluster 1, *P* < 0.05

^b^Compared to Cluster 2, *P* < 0.05

^c^Compared to Cluster 3, *P* < 0.05

^d^Compared to Cluster 4, *P* < 0.05

### Prevalence of obesity

**Fig. 2:**
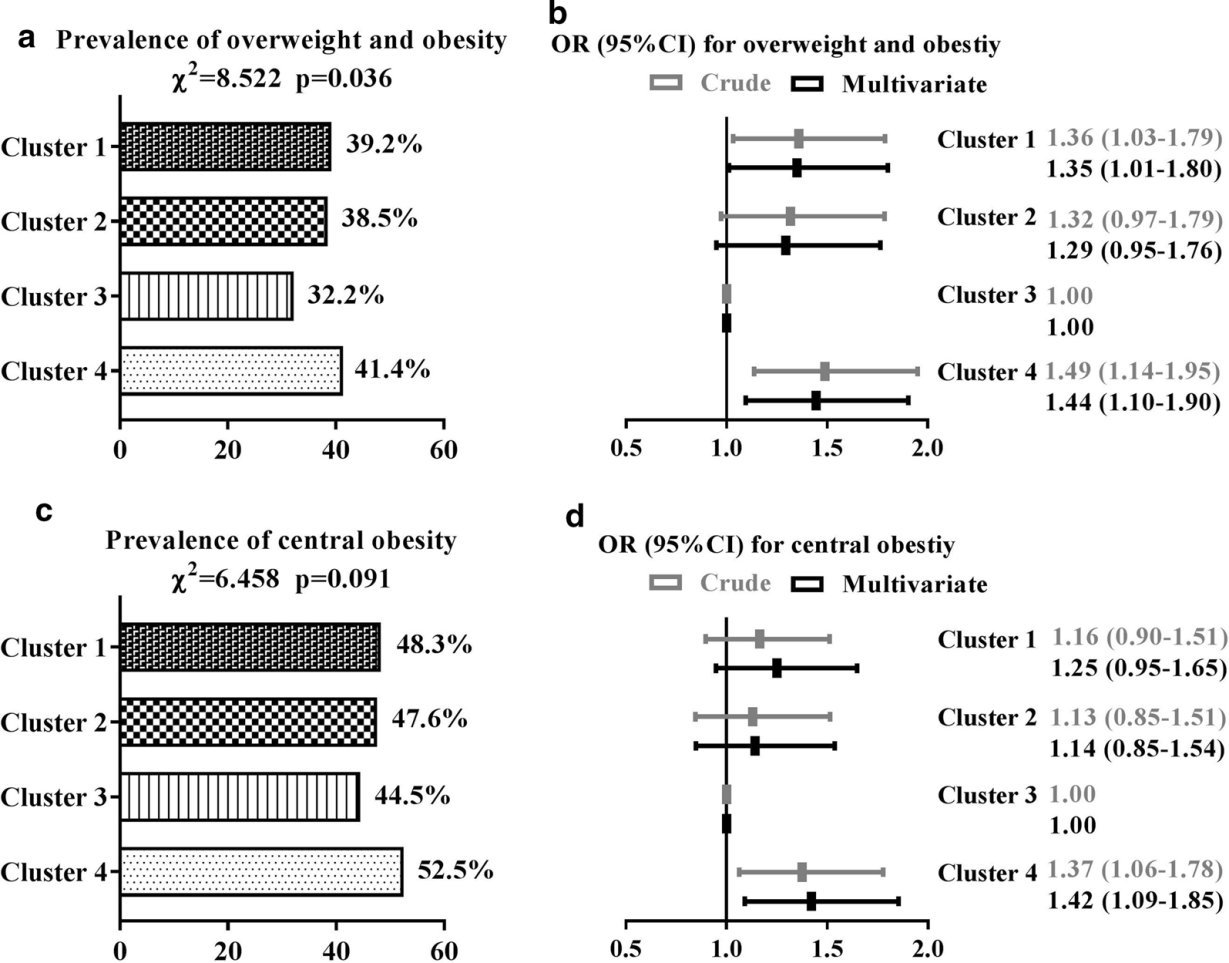
Prevalence of obesity among the participants and adjusted ORs (95% CIs) by cluster based on a logistic regression analysis adjusted for advanced age (≥ 65 years or no), sex (male or female), regular exercise (yes or no), current smoking status (yes or no), current drinking status (yes or no), and diabetic family history (yes or no). Overweight and obesity were defined as a BMI ≥ 24.0 kg/m^2^; central obesity was defined as a waist circumference ≥ 85.0 cm for men or ≥ 80.0 cm for women

Among the 2029 individuals, 783 were diagnosed with overweight and obesity by BMI, and 992 were diagnosed with central obesity by waist circumference, resulting in a prevalence of 38.6% and 48.9%, respectively. The lowest prevalence of overweight and obesity was observed in cluster 3 (32.2%), with moderate GL intake of 93 (85–102)/1000 kcal. Compared to cluster 3, the risk of overweight and obesity both increased in cluster 1 (with the highest GL intake of 112 (107–119)/1000 kcal) and cluster 4 (with the lowest GL intake of 90 (82–96)/1000 kcal), with multivariable adjusted ORs (95% CIs) of 1.35 (1.01–1.80) and 1.44 (1.10–1.90), respectively. A similar trend of the prevalence of central obesity was observed across the four clusters. Compared to cluster 3, the risk of central obesity increased in cluster 4, with a multivariable adjusted OR (95% CI) of 1.42 (1.09–1.85) (Fig. 2).

### Prevalence of diabetes

**Fig. 3:**
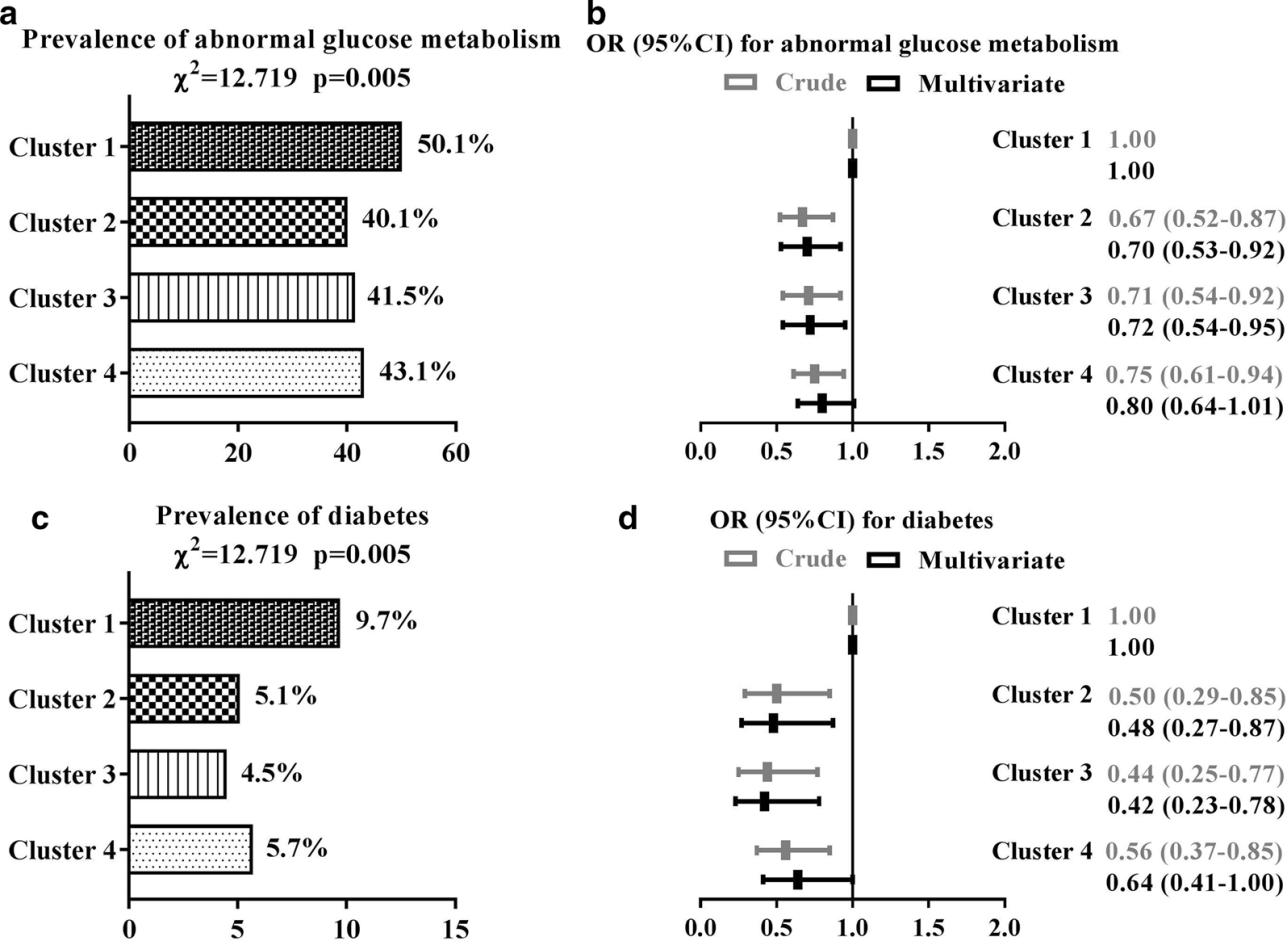
Prevalence of diabetes among the participants and adjusted ORs (95% CIs) by cluster based on a logistic regression analysis adjusted for advanced age (≥ 65 years or no), sex (male or female), regular exercise (yes or no), current smoking status (yes or no), current drinking status (yes or no), and diabetic family history (yes or no). Abnormal glucose metabolism was defined as FPG ≥ 6.1 mmol/L and/or 2-hPG ≥ 7.8 mmol/L; diabetes was defined as FPG ≥ 7.0 mmol/L and/or 2-hPG ≥ 11.1 mmol/L

Among the 2029 individuals, 901 were diagnosed with abnormal glucose metabolism, and 134 were diagnosed with diabetes by OGTT, resulting in a prevalence of 44.4% and 6.6%, respectively. The prevalence of both abnormal glucose metabolism and diabetes was relatively lower in clusters 2 and 3. Compared to cluster 1, the multivariable adjusted ORs (95% CI) of abnormal glucose metabolism and diabetes in cluster 2 were 0.70 (0.53–0.92) and 0.48 (0.27–0.87), and those in cluster 3 were 0.72 (0.54–0.95) and 0.42 (0.23–0.78), respectively (Fig. 3).

### The optimal GL range

Participants in cluster 1 consumed a typical high-carbohydrate/high-GL diet, with approximately 60% of the total energy derived from carbohydrates and 91% of carbohydrate derived from refined grains. In contrast, participants in cluster 4 consumed an improper low-carbohydrate/low-GL diet, with approximately 49% of the total energy derived from carbohydrates and 87% of carbohydrate derived from refined grains. The GLs of clusters 2 and 3 were slightly higher than those of cluster 4; however, the contribution rates of the macronutrients to the total energy were more consistent with the reference and only approximately 75% was derived from refined grains.

**Fig. 4:**
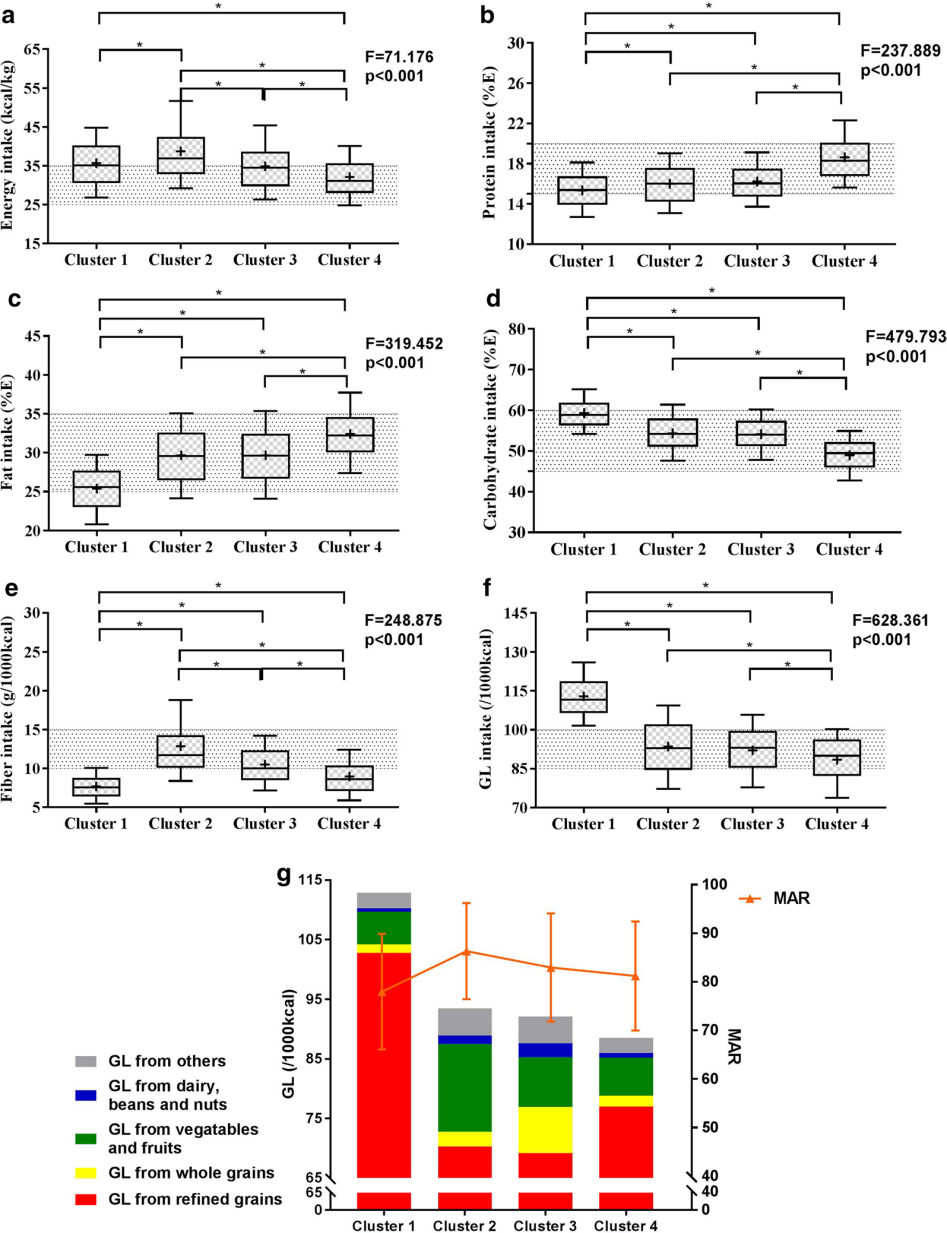
Dietary characteristics of the four identified clusters. Whisker-box plot with boxes indicating the median and 25th and 75th percentiles and whiskers indicating the 10th and 90th percentiles. “+” indicates the mean. The shadow indicates the Chinese dietary reference intakes (**a**–**e**) or the interquartile range of cluster 3 (**f**). *P < 0.05. *GL* glycemic load, *MAR* micronutrient adequacy ratio

The total GL was similar between clusters 2 and 3; however, the food composition differed. Cluster 2 consumed the highest GL intake from fruit and nuts, while cluster 3 consumed the highest GL intake from whole grains, mixed beans, dairy, beans and nuts. The MARs were higher in clusters 2 and 3 (Fig. 4).

Given the associations among the clusters, nutrient intakes, obesity and diabetes risk, the optimal GL range was determined to be the interquartile of clusters 2 and 3, which was (85–100)/1000 kcal with the reference intake of carbohydrate, fat, and protein and proper food intake.

## Discussion

Consistent with several previous cross-sectional studies [36, 37], our results suggest that a low GL is associated with better glucose homeostasis. Nevertheless, our results contributed to the debate regarding whether excessive GL restriction may increase the risk of obesity. In this study, participants with moderate GL intake (clusters 2 and 3) had a lower prevalence of overweight and obesity, while both those with the highest GL intake (cluster 1) and the lowest GL intake (cluster 4) showed an increased risk of overweight and obesity. Only one previous study suggested a negative association between GL and BMI [38], while other studies have indicated that GL is not associated with the BMI [37, 39, 40]. However, in addition to BMI, associations with waist circumference have been examined, and both a positive association [41] and no association [39, 40] between GL and waist circumference have been reported. Based on our results, we considered both obesity and diabetes risk and nutrient intake and showed that the optimal intake, which is preliminarily set at (85–100)/1000 kcal, is better than the lowest GL.

In the present study, dietary GI and GL were assessed using a previously validated 3-day food record instead of an FFQ. This methodology was selected for three reasons. First, FFQ usually overestimates food intake compared to other nutritional assessment methods, which leads to an overestimation of the energy and nutritional values of diets [42]. Second, possible errors include the omission or addition of food, as well as an inadequate assessment of the frequency and amount of consumed products [43]. Third, with a 3-day food record, details about the sources, preparation, and processing of foods and timing and location of meals together with quantitative data on all food sources of energy and nutrients can be captured. Last, a 3-day food record can be designed to be culturally sensitive and cognitively easy, making it especially suitable for respondents with limited education, such as elderly adults [44]. In addition, in our study, the 3-day food record is a prospective method that is independent of the participant’s memory and covers three consecutive days. Therefore, the food record provides relatively accurate data concerning the intake of food and nutrients. In our reproducibility and validity test, the intake of certain foods was sometimes underestimated using 3-day food records. However, the intake of cereal, which is the dominant source of GL, rarely changed.

Dietary pattern analyses using component [45, 46] or cluster analyses [47, 48] reflecting the complexity of dietary intake have recently received greater attention from nutritional epidemiologists [49–51]. Component analyses reduce the number of variables by identifying independent vectors that are combinations of original correlated variables; cluster analyses create groups or clusters of subjects with similar profiles and are very useful for descriptive purposes. In this study, we preliminarily used a cluster analysis to identify the GL intake patterns, and nonoverlapping groups of individuals who exhibited similar patterns of GL intake were created based on the dominant pattern of GL intake. To the best of our knowledge, there are no comparable studies investigating GL clusters in terms of overweight and obesity or diabetes.

Traditionally, studies investigating dietary GL intake and chronic metabolic disease have focused on the total GL. However, food is typically consumed in combination, not in isolation, and therefore, comprehensive investigations are needed to understand the dietary patterns associated with a lower risk of diabetes. Dietary GL decreased from cluster 1 to cluster 4. However, the lowest risks for overweight and obesity, central obesity, abnormal glucose metabolism, and diabetes were observed in the middle clusters (cluster 2 or 3) rather than either the highest (cluster 1) or the lowest (cluster 4) cluster. Considering the food sources of the GL, although cluster 4 consumed the lowest total GL, approximately 87% of the GL was derived from refined grains, which seemed to increase the risk of type 2 diabetes [7]. In contrast, the total GLs in clusters 2 and 3 were slightly higher than those that in cluster 4; however, approximately 75% of both were derived from refined grains. Cluster 2 consumed the highest intake of GL from fruit and nuts, and cluster 3 consumed the highest intake of GL from whole grains and mixed beans, dairy and beans. Numerous previous studies have suggested the favorable effects of such foods on obesity and diabetes [7].

Dietary patterns (i.e., the macronutrient ratios and sources) impact the inflammatory potential and obesity or diabetes risk. Compared to the Chinese dietary reference intakes of macronutrients, participants with moderate GL intake (clusters 2 and 3) were more consistent with the macronutrient intake reference. In contrast, participants with the lowest GL intake (cluster 4) consumed relatively higher fat and protein. Generally, accepted that consuming a high fat diet increases the likelihood of obesity, which is one of the identified significant risk factors for diabetes. However, the role of proteins in diabetes prevention is conflicting. Dietary proteins have an insulinotropic effect and promote insulin secretion, which leads to an increased rate of glucose clearance from the blood [52]. However, the results from clinical trials and observational studies have been mixed. A meta-analysis showed beneficial effects of a high-protein diet on several obesity and cardiometabolic parameters, including weight loss and fasting insulin [53]. Conversely, several large prospective cohort studies have shown detrimental associations between protein intake and diabetes risk [54, 55]. A meta-analysis suggested that high total protein and animal protein intake were associated with an increased risk of diabetes while high plant protein intake was associated with a decreased risk [56]. Therefore, the efficacy and safety of high-protein, low-carbohydrate diets have to be studied more extensively.

The relationship between individual micronutrients and a low-GL diet is still uncertain. Low-GI foods are by definition moderate to high sources of carbohydrates, yet some are also particularly rich in micronutrients, such as fruits, whole grains and dairy products. Several studies have reported that a low-GL diet is associated with higher intakes of micronutrients [57], whereas a diet with low or no gluten may lead to micronutrient deficiencies [58]. Combined with our results, a low GL with a proper food intake diet, which ideally contains many whole grains, mixed beans, vegetables, fruits, dairy, nuts and beans, should be fundamental for the adequate intake of micronutrients. A reasonable collocation dietary pattern could be better than a dietary pattern that excessively restricts the GL.

Our study has the following limitations. First, a cross-sectional design and convenience sampling were used such that the majority of the study subjects were women (69%). Therefore, sex (male or female) was adjusted when analyzing the association between dietary GL and the prevalence of abnormal glucose metabolism. In addition, the data analyzed in this cross-sectional analysis were derived from a baseline survey of an ongoing multiethnic, epidemiological study. Therefore, the results could be further studied based on the following prospective observations. Second, all participants were Chinese with traditional high-GL dietary habits. The generalization of the results to other ethnic groups should be performed with caution. Third, measurements of dietary intake were secured by self-reported dietary records, as known recovery biomarkers of GL are limited. To secure a securing more accurate measurement of diet, all participants were trained on how to record the diet intake and were suggested to record at the time of the eating occasion. Despite these limitations, this study was the first to evaluate the associations among the GL, macro- and micronutrient intake and the risk of obesity and diabetes. In addition, the optimal range of the GL for lowering both obesity and diabetes risk was preliminarily explored.

## Conclusion

Our results demonstrate that reducing GL to prevent diabetes deserves more attention based on dietary patterns. An appropriate GL is better for reducing the risk of obesity and diabetes than excessive GL restriction. This study underscores that required educational interventions should not only promote a specific GL limitation but also promote a more general healthy eating pattern.

## Data Availability

The datasets used in the present study are available from the corresponding author upon reasonable request.

## Abbreviations

2-hPG: 2-Hour plasma glucose
BMI: Body mass index
CI: Confidence interval
DF: Dietary fiber
FPG: Fasting plasma glucose
GL: Glycemic load
GI: Glycemic index
IBW: Ideal body weight
KMO: Kaiser–Meyer–Olkin
MET: Metabolic equivalent
OGTT: Oral glucose tolerance test
OR: Odds ratio
RCM: Rotated component matrix
